# Supplementary material for: A manifold-based framework for studying the dynamics of the vaginal microbiome
Source: NPJ Biofilms Microbiomes. 2023 Dec 15;9:102. doi: 10.1038/s41522-023-00471-8 (PMC10724123; doi:10.1038/s41522-023-00471-8)
Supplement: Supplementary file 1 — Supplementary Information [file 41522_2023_471_MOESM1_ESM.pdf]

Supplementary Information

A Manifold-Based Framework for Studying the Dynamics of the Vaginal Microbiome

Mor Tsamir-Rimon and Elhanan Borenstein

Supplementary figures

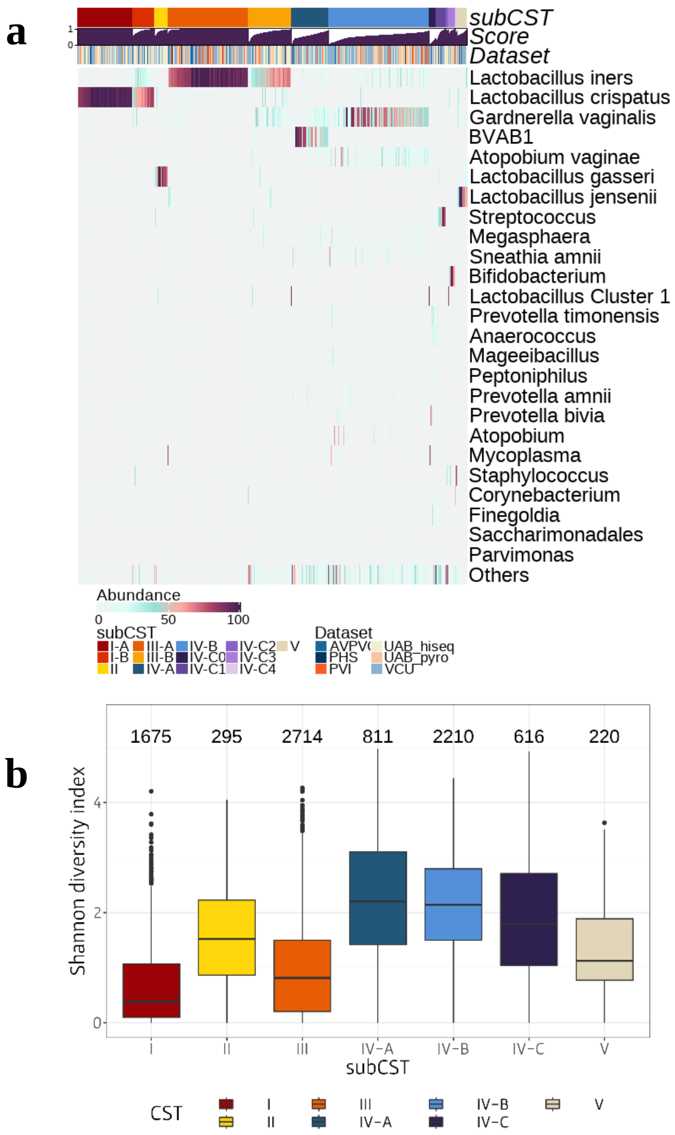

Supplementary Figure 1: (a) Heatmap representation of the abundances of the 25 most prevalent bacterial taxa, where each column displays a sample and each row displays a specific bacterial taxon. The first rows of the heatmap indicates other characteristics of each sample. In the first row, each color represents the dataset the sample was obtained from. The second row's colors represent the sample's subCST assignment (as previously defined[5]). In the third row, the height of the bar represents the similarity score of the samples composition to core CST defined by France et al. (see Methods). **(b) Box plot displays the average of Shannon diversity index for each CST**. Above each box displayed the number of samples assigned to this CST.

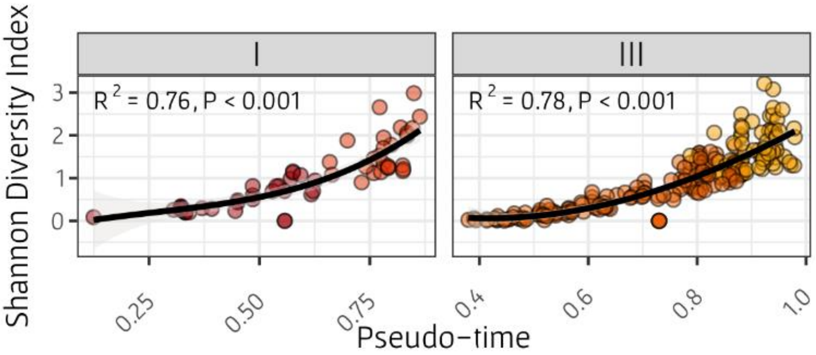

Supplementary Figure 2: Shannon diversity index as a function of pseudo-time label of 515 held-out samples. Pseudo-time label was assigned to each sample by its nearest neighbor on the manifold. Each plot represents a different CST arm, and colors indicate subCST assigned for each sample. The slope was determined using linear regression to transformed polynomial values of pseudo-time. The R^2 and p-value presented in each panel were determined by Spearman correlation test. CST II and V are omitted due to each having only one held-out sample assigned.

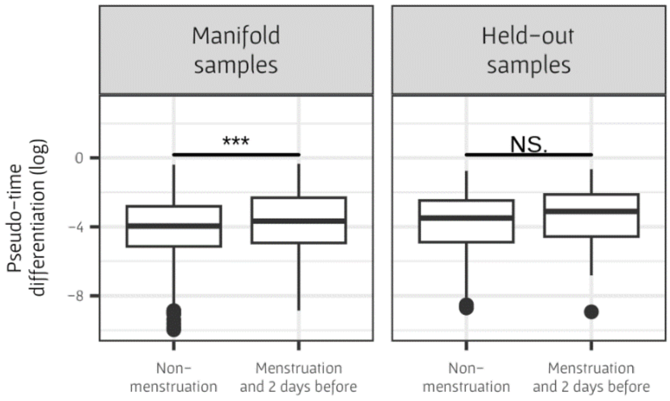

Supplementary Figure 3: Box plots of pseudo-time difference from previous sample in log scale between samples in different menstruation status. Menstruation relates to samples from two days before menstruation period and menstruation period samples. Right panel represents results of samples from manifold analysis (including 2292 samples) and right panel represents results of held-out samples (including 348 samples). Results of Wilcoxon tests are presented on top of each plot, where three asterisks indicate P. value under 0.001, two asterisks indicate P. value under 0.01, one asterisk indicates P. value under 0.05, and NS indicate non-significant.

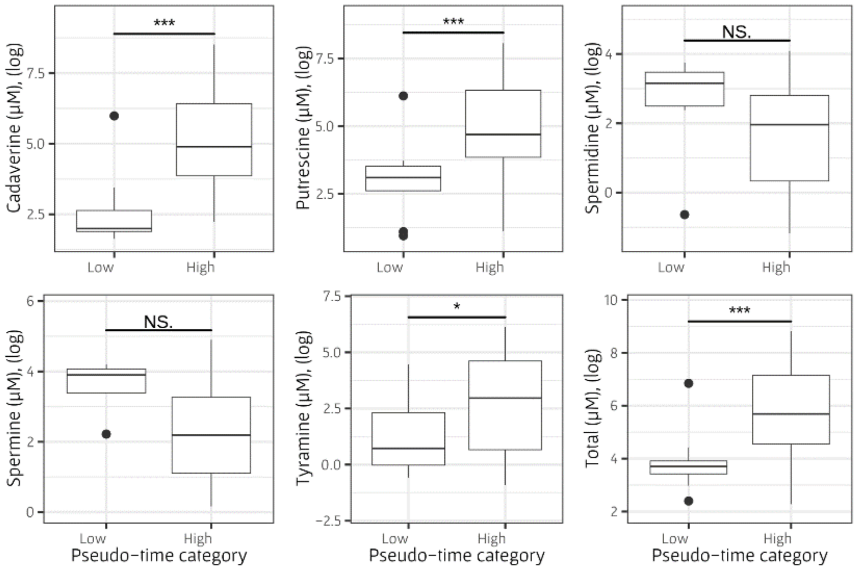

Supplementary Figure 4: Box plots of five biogenic amines levels in log scale: cadaverine, putrescine, spermine, spermidine, tyramine, and total sum of all metabolites, in high pseudo-time (>0.9) and low pseudo-time. Results of Wilcoxon test are presented on top of each plot, where three asterisks indicate P. value under 0.001, two asterisks indicate P. value under 0.01, one asterisk indicates P. value under 0.05, and NS indicate non-significant. The analysis was performed on 73 samples with available metabolite data.

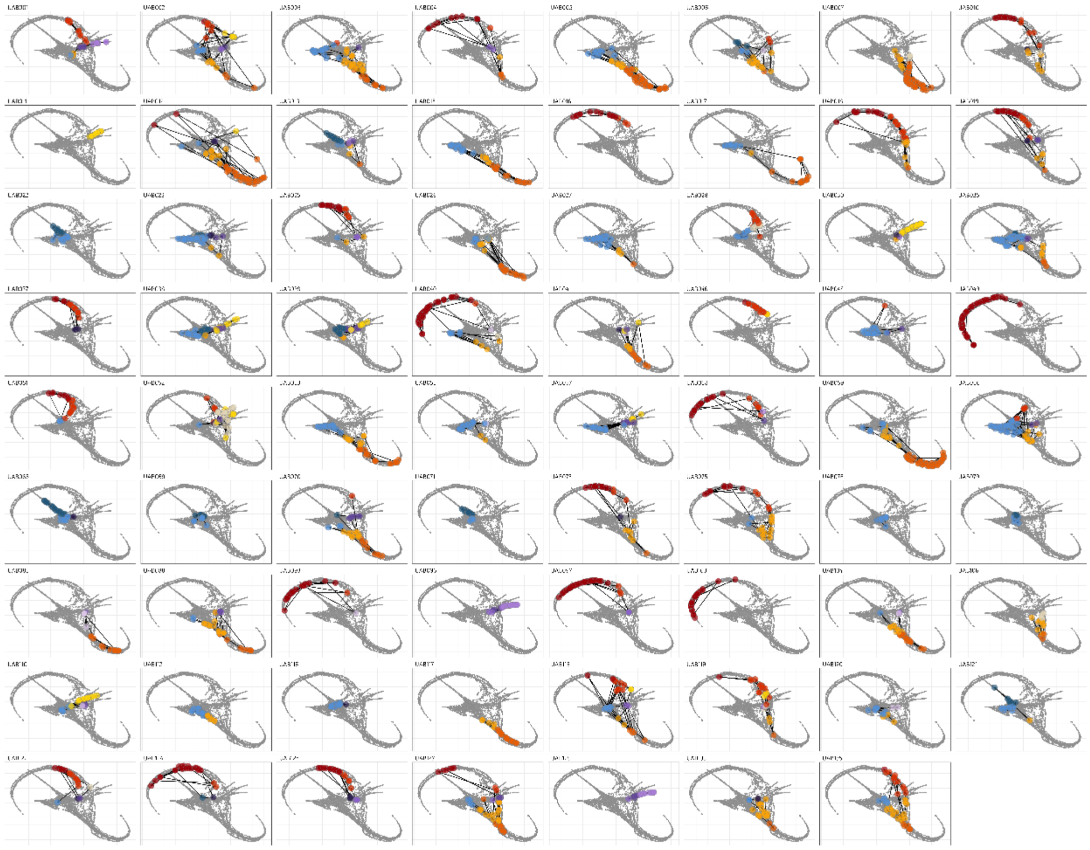

Supplementary Figure 5: Trajectories of 71 women from the longitudinal dataset (UAB) which were included in the manifold analysis. Each plot represents a different woman's trajectory. Grey dots represent the UMAP visualization of the vaginal microbiome composition manifold, and the colored dots represent the locations of samples obtained from a specific woman, where each color represent the determined subCST of the sample. Black arrows display the direction of the woman's trajectory, showing the order of the samples based on the time they were obtained in the experiment.

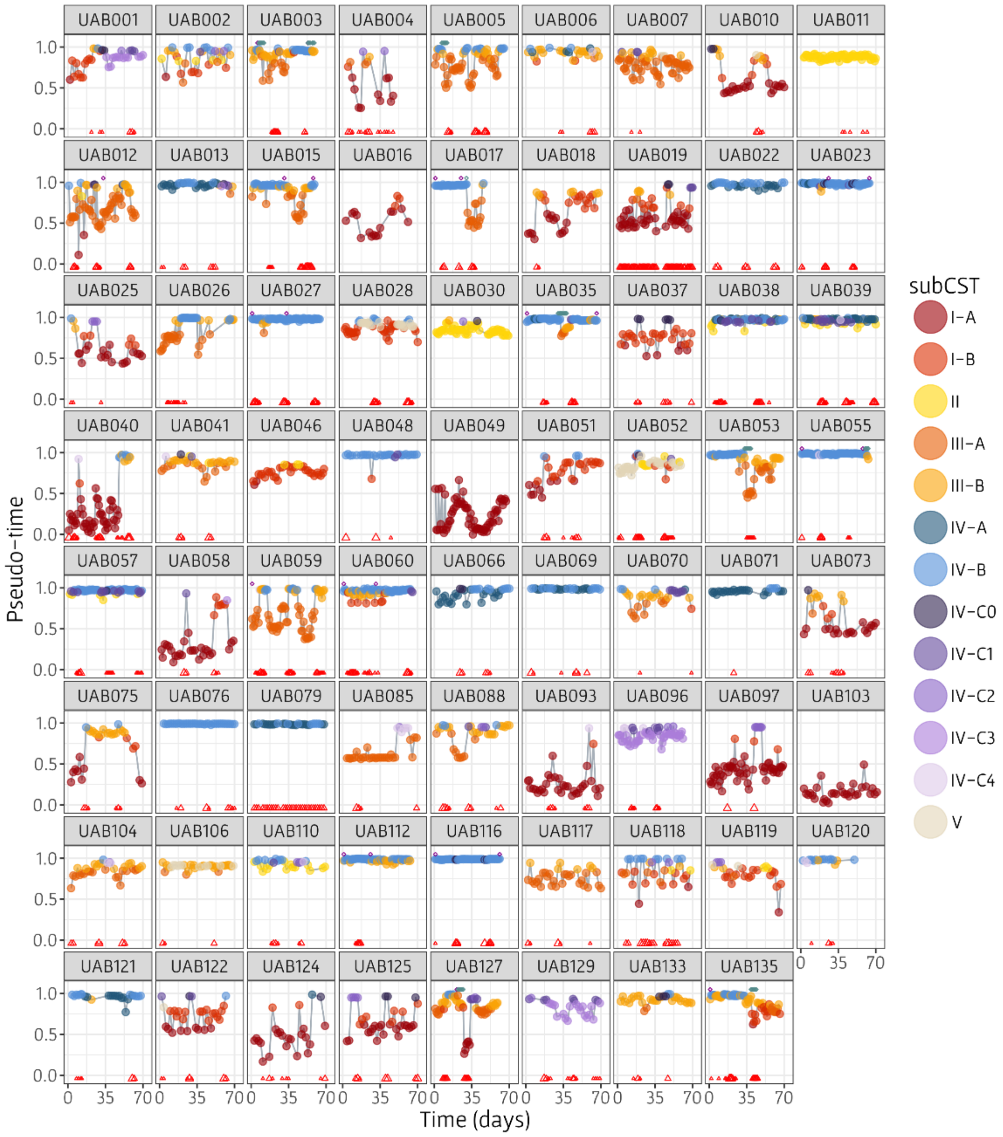

Supplementary Figure 6. Pseudo-time label to chronological time-point of each woman, representing her trajectory. Higher value of pseudo-time label indicates it's close to root samples (i.e., BV state), while lower value of pseudo-time indicates it's distant from root samples. Dot's colors represent the sample's subCST. Red triangles in the bottom of each plot represent self-reported menstruation, where size indicates menstruation spotting (small), medium or heavy bleeding (large). Purple diamonds of the top of each plot represent time in which that sample was diagnosed with positive Amsel's criteria (with or without self-reported symptoms), and turquoise diamonds represent time with BV-medication use.

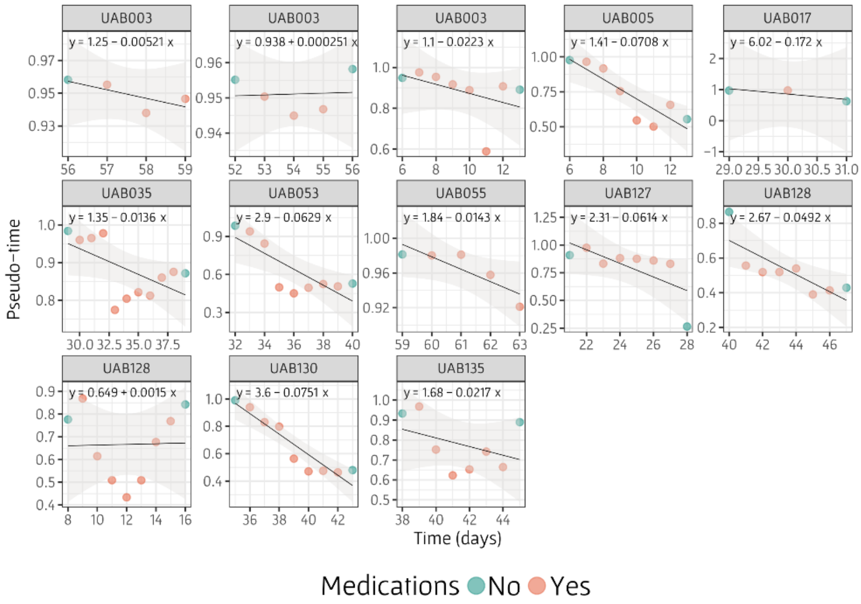

Supplementary Figure 7. Pseudo-time as a function of time in days with BV medication use, as well as the day before and after treatment. Red points represent samples where the woman has reported medication use and green points represent the days before and after treatment. To assess the direction of pseudo-time progression, we determined the slope of each plot by employing a linear regression model equation using the `stat_poly_eq` function from the *ggpmisc* package in R. Linear model equation is presented in each top right plot, while linear regression model is represented by black lines.

Supplementary Tables

Supplementary Table 1: Correlation between Shannon diversity index and pseudo-time, calculated for each dataset separately.

Dataset	CST	R^2	P value
UAB (Illumina Hiseq)	I	0.821	4E-216
	II	0.815	1E-63
	III	0.780	1E-113
	V	0.219	0.0325
UAB (Pyrosequencing)	I	0.927	1E-82
	II	0.945	0.0004
	III	0.826	5E-174
	V	0.725	1E-11
PVI	I	0.850	5E-10
	III	0.880	4E-92
AVPVC	I	0.735	4E-24
	II	0.954	6E-11
	III	0.871	9E-64
	V	0.922	4E-10
VCU	I	0.802	9E-256
	II	0.607	3E-16
	III	0.808	0
	V	0.761	4E-33
PHS	I	0.835	1E-10
	III	0.846	5E-30
All	I	0.786	0
	II	0.655	9E-67
	III	0.803	0
	V	0.688	5E-54

Supplementary Table 2: Statistical analysis results of BV indicators levels in comparison to pseudo-time value

Parameter	CST	# of samples (# position samples)	P value	FDR
Nugent (Spearman)	I	683	9.9E-24	3.5E-22
	II	183	0.0009	0.00285
	III	554	1.7E-20	2E-19
	IV-A	246	1.5E-05	5.1E-05
	IV-B	346	3.2E-06	1.2E-05
	IV-C	188	0.03193	0.05881
	V	60	0.01031	0.02255
Amsel's test (Wilcoxon)	I	209 (3)	0.00622	0.01452
	III	747 (61)	1.4E-12	1E-11
	IV-A	79 (55)	0.0117	0.02409
	IV-B	771 (153)	1E-10	6E-10
	IV-C	124 (6)	0.05543	0.08818
pH (Spearman)	I	870	0.0532	0.08818
	II	210	0.51537	0.56369
	III	1214	8.5E-23	1.5E-21
	IV-A	290	0.20754	0.26903
	IV-B	1051	0.00597	0.01452
	IV-C	315	0.00156	0.00456
	V	105	0.39125	0.47219
Clue cells (Wilcoxon)	I	59 (2)	0.07505	0.11421
	III	282 (26)	2.8E-08	1.4E-07
	IV-A	72 (39)	0.51446	0.56369
	IV-B	236 (82)	0.14464	0.1947
	IV-C	26 (3)	0.00538	0.0145
	V	2 (1)	1	1
Whiff test (Wilcoxon)	I	59 (2)	0.031	0.05881
	III	282 (42)	3.5E-15	3.1E-14
	IV-A	72 (46)	0.12959	0.18847
	IV-B	236 (106)	0.13462	0.18847
	IV-C	26 (6)	0.83577	0.88643
	V	2 (1)	1	1
Vaginal fluid (Wilcoxon)	I	56 (3)	0.03807	0.06662
	III	276 (48)	2.3E-07	1E-06
	IV-A	71 (42)	0.47435	0.55341
	IV-B	235 (50)	0.3876	0.47219

Each BV indicator was compared to pseudo-time value in each CST arm separately. Binary BV indicators associations were calculates using Wilcoxon test (with the number of positive samples in each CST shown in the table in parentheses), while continuous BV indicators associations were calculated using Spearman correlation. Multiple comparisons correction was conducted using FDR correction.
